# Bone fracture as a novel immune‐related adverse event with immune checkpoint inhibitors: Case series and large‐scale pharmacovigilance analysis

**DOI:** 10.1002/ijc.33592

**Published:** 2021-05-04

**Authors:** Daria Maria Filippini, Milo Gatti, Vito Di Martino, Stefano Cavalieri, Michele Fusaroli, Andrea Ardizzoni, Emanuel Raschi, Lisa Licitra

**Affiliations:** ^1^ Head and Neck Medical Oncology Unit Fondazione IRCCS—Istituto Nazionale dei Tumori Milan Italy; ^2^ Department of Oncology and Hemato‐Oncology University of Milan Milan Italy; ^3^ Medical Oncology Unit, Department of Experimental, Diagnostic and Specialty Medicine Policlinico S. Orsola‐Malpighi, Alma Mater Studiorum—University of Bologna Bologna Italy; ^4^ Pharmacology Unit, Department of Medical and Surgical Sciences Alma Mater Studiorum—University of Bologna Bologna Italy

**Keywords:** disproportionality analysis, immune checkpoint inhibitors, skeletal immune‐related adverse events, vertebral fracture

## Abstract

Although immune checkpoint inhibitors (ICIs) are associated with different immune‐related adverse events (irAEs), the potential effect on the skeleton is poorly defined albeit biologically plausible and assessable through pharmacovigilance. We described a case series of patients experiencing skeletal fractures while on ICIs at the National Cancer Institute of Milan. To better characterize the clinical features of skeletal irAEs reported with ICIs, we queried the FDA Adverse Event Reporting System (FAERS) and performed disproportionality analysis by means of reporting odds ratios (RORs), deemed significant by a lower limit of the 95% confidence interval (LL95% CI) > 1. Bone AEs emerging as significant were scrutinized in terms of demographic and clinical data, including concomitant irAEs or drugs affecting bone resorption or causing bone damage. Four patients with skeletal events while on ICIs were included in our case series, of which three exhibited vertebral fractures. In FAERS, 650 patients with bone and joint injuries and treated with ICIs were retrieved, accounting for 822 drug‐event pairs. Statistically significant ROR was found for eight, two and one bone AEs respectively with PD‐1, PD‐L1 and CTLA‐4 inhibitors, being pathological fracture (N = 46; ROR = 3.17; LL95%CI = 2.37), spinal compression fracture (42; 2.51; 1.91), and femoral neck fracture (26; 2.38; 1.62) the most common. Concomitant irAEs or drugs affecting bone metabolism were poorly reported. The increased reporting of serious vertebral fractures in patients without concomitant irAEs and no apparent preexisting risk factors could suggest a possible cause‐effect relationship and calls for close clinical monitoring and implementation of dedicated guidelines.

## Abstract 2

What's new?

Immune checkpoint inhibitors (ICIs), while potentially improving cancer patient survival, are associated with sometimes severe immune‐related adverse events. While these events affect all host tissues, little is known about their impact on bone in particular. Here, the authors investigated accounts of bone and joint injuries among patients taking ICIs, using clinical data and reports submitted to a pharmacovigilance database. Analyses show that ICIs may precipitate adverse skeletal events and reveal an increased reporting of serious vertebral fractures in patients lacking pre‐existing risk factors. The findings suggest that risk stratification and monitoring for skeletal lesions in patients taking ICIs is warranted.

**Abbreviations**
AE: adverse event
BMI: body mass index
CTLA‐4: cytotoxic T‐lymphocyte antigen 4
eGFR: estimated glomerular filtration rate
FAERS: FDA Adverse Event Reporting System
HLGT: High Level Group Term
ICI: immune checkpoint inhibitor
IQR: interquartile range
irAE: immune‐related adverse event
LL95% CI: lower limit of the 95% confidence interval
PD‐1: programmed cell death 1
PD‐L1: programmed cell death ligand 1
PPI: proton pump inhibitor
PT: preferred term
PTH: parathyroid hormone
Q1: first quarter
ROR: reporting odds ratio

## 1 INTRODUCTION

The advent of immune checkpoint inhibitors (ICIs) has markedly improved patient survival in different subtypes of metastatic cancer, by enhancing cytotoxic T‐cells activity through blocking either cytotoxic T‐lymphocyte antigen 4 (CTLA‐4) or programmed cell death 1 (PD‐1) or its ligand (PD‐L1).^1^ However, ICIs are associated with a variety of immune‐related adverse events (irAEs), virtually affecting all host tissues, most of which have been described through pharmacovigilance analyses.^2^, ^3^, ^4^, ^5^, ^6^ The effect on the skeleton is poorly studied and, to the best of our knowledge, only a small case series exists, including three patients with new‐onset osteoporosis leading to fracture.^7^

**FIGURE 1:**
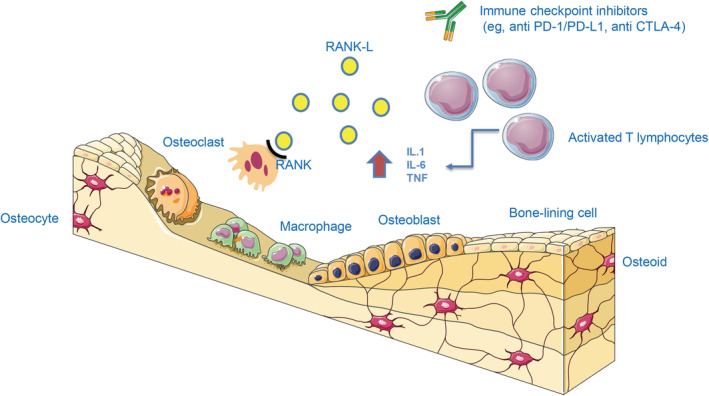
Potential mechanisms leading to bone loss under treatment with immune checkpoint inhibitors (see text for details). Created through a public service (https://smart.servier.com/) [Color figure can be viewed at wileyonlinelibrary.com]

This report stems from our experience at the National Cancer Institute Research Center, in Milan, where different cases of suspected ICI‐related bone fractures occurred in patients affected by head and neck cancer. This prompted us to investigate the potential biological rationale subtending our findings. Emerging evidence suggests that systemic activation of T cells in vivo leads to an osteoprotegerin ligand‐mediated increase in osteoclastogenesis and bone loss (Figure 1). In fact, ICIs can enhance bone resorption by activating T cells,^8^ which in turn causes bone loss with bone fragility, increasing the risk of fractures.^9^, ^10^

In the recent past, the Food and Drug Administration Adverse Event Reporting System (FAERS) has attracted considerable interest among clinicians for accurate and timely characterization of drug‐related risks occurring in real‐world cancer patients with comorbidities and polypharmacotherapy. These postmarketing studies are particularly suited to early detect rare, unexpected and delayed adverse events (AEs), which cannot be fully appreciated in pivotal trials (where only irAEs occurring in at least 5% of patients were reported), and are recommended for real‐time safety assessment of recently marketed drugs receiving accelerated regulatory approval.^11^

On these grounds, we aimed to describe spectrum and clinical features of ICI‐related skeletal lesions by retrospectively analyzing two real‐world settings: clinical data of patients admitted to the National Cancer Research Center in Milan (case series) and unsolicited reports submitted to the worldwide FAERS (pharmacovigilance database analysis).

## 2 MATERIAL AND METHODS

### 2.1 Clinical data collection (case series)

Four patients treated with ICIs at the National Cancer Institute of Milan (reference center for the management of head and neck cancers) experienced bone fracture. Patient and tumor features including medical history (including risk factors for bone loss, namely preexisting osteoporosis, tobacco or alcohol abuse, chronic renal disease and prolonged corticosteroid use), tumor histology, systemic cancer therapies, sites of fracture, time to onset, laboratory and radiological findings, occurrence of irAEs or other AEs related to ICIs, and the use of concomitant medications were collected from the review of medical records.

### 2.2 Case and exposure definition in pharmacovigilance analysis

As of March 31, 2020, FAERS collected more than 20 million reports and covered virtually worldwide population (relevant catchment area includes also serious reports from EU and other non‐US countries). We queried the FAERS database (public dashboard) to identify all reports recorded between the first quarter (Q1) of 2004 and Q1 of 2020. We searched all the 112 preferred terms (PTs) listed in “Bone and joint injuries” High Level Group Term (HLGT), and PTs concerning osteonecrosis (namely “*osteonecrosis*,” “*osteonecrosis of jaw*” and “*osteonecrosis of external auditory canal*”), classified according to the Medical Dictionary for Regulatory Activities. Furthermore, the event “fall” was searched as negative control, in order to verify whether skeletal toxicity is indirectly related to trauma.

Different groups of exposure of interest were considered, including anti‐CTLA‐4 (ipilimumab, tremelimumab), anti‐PD‐1 (nivolumab, pembrolizumab, cemiplimab) and anti‐PD‐L1 (atezolizumab, avelumab, durvalumab). In our study, exposure assessment was defined when ICIs were recorded as suspect.

### 2.3 Disproportionality analysis

As a measure of disproportionality, we calculated the reporting odds ratio (ROR) with relevant 95% confidence interval (CI); statistical significance was defined by a lower limit of the 95% CI of the ROR exceeding 1, with at least 5 cases reported, to reduce the likelihood of false positives.^5^ Specifically, a case‐noncase approach was applied: cases were defined by “bone and joint injuries” reports recorded for ICIs, while noncases were represented by AE reports recorded for all other drugs in FAERS. The ROR is the odds of exposure to ICIs among the cases divided by the odds of exposure to ICIs among the noncases. If the proportion of the AE of interest is greater in patients exposed to ICIs (cases) than in patients exposed to all other drugs reported in FAERS (noncases), a disproportionality signal emerges.^12^, ^13^ Cases counted as many‐fold as the number of “bone and joint injuries” events identified by relevant PTs recorded in a given report.

### 2.4 Clinical characterization of disproportionality signals

Skeletal AEs emerging from disproportionality analysis were further scrutinized to remove potential duplicates (ie, records overlapping in at least three out of four key fields: event date, age, sex, and reporter's country.

Remaining cases were described in terms of clinical features, including potential existence of confounders: demographic information (age, gender, reporter country), seriousness (ie, those resulting in death, hospitalization—initial or prolonged—life‐threatening events or leading to disability or congenital anomalies), fatality rate (ie, proportion of death reports), therapeutic regimen and indication, concomitant bone metastases, concomitant endocrine irAEs (proxy for occurrence of secondary osteoporosis caused by calcium metabolism disorders, hypogonadism, endogen excess of glucocorticoids or requirement for steroid therapy), proportion of falls and myositis, and concomitant neurological AEs.

Additionally, concomitant drugs were analyzed by searching for proton pump inhibitors (PPIs), suggested to increase the risk of skeleton fracture,^14^ agents acting on bone resorption (ie, bisphosphonates, denosumab, teriparatide, as a proxy of preexisting osteoporosis) or causing bone damage (ie, corticosteroids, antiepileptics, antihormonal agents) based on the list proposed by Nguyen et al.^15^

**FIGURE 2:**
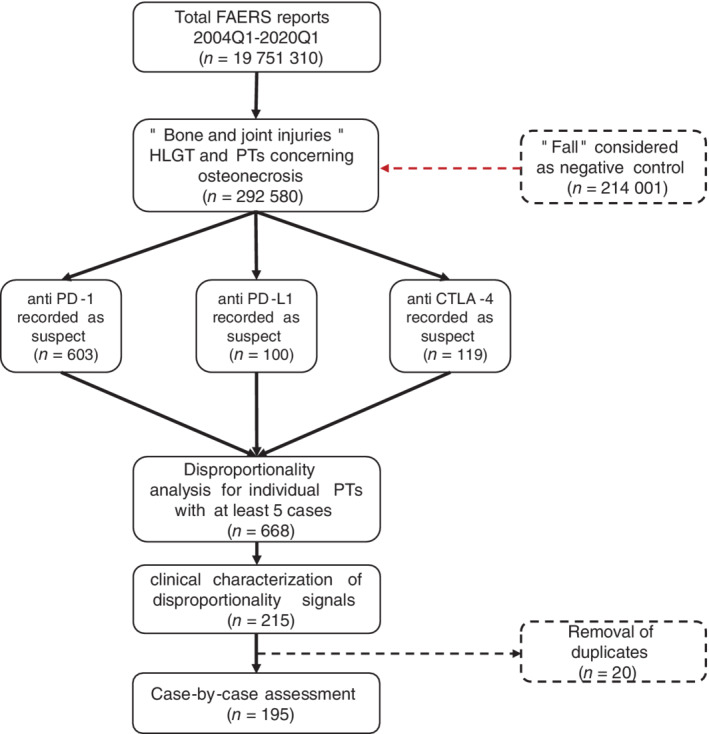
Flowchart showing the implemented methodological steps for the extraction and analysis of the FDA Adverse Reporting System (FAERS) data [Color figure can be viewed at wileyonlinelibrary.com]

Finally, latency of the skeletal events was calculated as the difference between the start of therapy and the date the event occurred (median days with interquartile range—IQR). To avoid the potential confounding factors of concomitant nonskeleton irAEs, the onset was calculated only for cases in which events of interest were reported alone (ie, without concomitant irAEs). The flowchart of methodological steps followed for analysis of FAERS is showed in Figure 2.

## 3 RESULTS

### 3.1 Case series

Four patients developed new osteoporotic fractures while on systemic treatment with ICIs administered alone or in combination (Table 1). Patients were 62 to 70 years old at the time of development of the skeletal event, and three were females. Three patients experienced vertebral fractures and one had a calcaneal fracture. The time to onset was 2.5 to 15.5 months. None of the patients suffered from osteoporosis/osteopenia. One patient had primitive hyperparathyroidism treated with calcium modulating drug (cinacalcet), the serum calcium level was 10.96 mg/dL (n.v. 8.6‐10.20) and serum parathyroid hormone (PTH) was 184 pg/mL (n.v. 15‐65), at the time of the skeletal event. Three patients were former smokers (<40 pack/years) while one patient was a nonsmoker. None of the patients had a history of alcohol abuse. Two patients had mild renal failure (eGFR 60‐90 mL/min/1.73 m^2^). Notably, all four patients were on treatment with PPIs for more than 1 year. None of the patients was treated with long‐term corticosteroids. No other known drugs causing bone loss were reported. None of the four patients had experienced additional irAE not related to the musculoskeletal system. Interestingly, one patient experienced a clinical tumor complete response after 3 months from the bone fracture.

**TABLE 1:** Case series: patient demographics and clinical data

							Concomitant drugs				
Patient	AE	Age	Sex	BMI	ICIs	Therapeutic indications	Agents acting on bone resorption	Agents causing bone damage	PPIs >1 y	Endocrine IrAEs	Metastases to bone
1	Dorsal vertebral (D12) fracture	69	F	24.5	Anti‐PD‐1	Recurrent cutaneous SCC	None	None	PAN	None	None
2	Calcaneal fracture	62	F	17.9	Anti‐PD‐L1	Metastatic HPV positive OPC SCC	None	None	PAN	None	None
3	Lumbar vertebral (L1) fracture	70	M	17.41	Anti‐PD‐L1	Recurrent Hypopharingeal SCC	None	None	LAN	None	None
4	Multiple vertebral (D7‐L5) fractures	70	F	20.25	Anti‐PD‐1	Recurrent OC SCC	None	None	PAN	Primitive hyperpara‐thyroidism	None

Abbreviations: AE, adverse event; BMI, body mass index; HPV, human papillomavirus; ICIs, immune checkpoint inhibitors; IrAEs, immune‐related adverse events; LAN, lansoprazole; OC, oral cavity; OPC, oropharyngeal cancer; PAN, pantoprazole; PPIs, proton pump inhibitors; SCC, squamous cell carcinoma.

### 3.2 Data from the FAERS analysis

**FIGURE 3:**
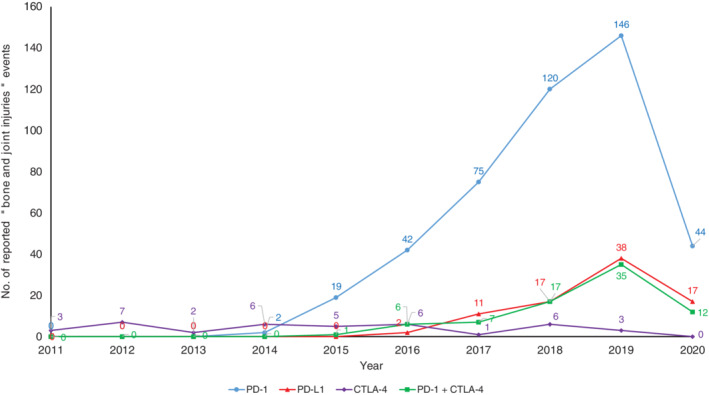
Time trends of reports concerning “bone and joint injuries” events with immune checkpoint inhibitors in FDA Adverse Reporting System (FAERS). PD‐1 total reports: 2011—0; 2012—0; 2013—0; 2014—494; 2015—3298; 2016—8248; 2017—11 555; 2018—14 833; 2019—18 282; 2020 (Q1)—6775. PD‐L1 total reports: 2011—0; 2012—0; 2013—0; 2014—18; 2015—45; 2016—348; 2017—1390; 2018—2784; 2019—4626; 2020 (Q1)—1935. CTLA‐4 total reports: 2011—497; 2012—1182; 2013—1042; 2014—1479; 2015—1920; 2016—1981; 2017—3047; 2018—3380; 2019—4465; 2020 (Q1)—2026. CTLA‐4, cytotoxic T‐lymphocyte antigen 4; PD‐1, programmed cell death 1; PD‐L1, ligand of programmed cell death 1 [Color figure can be viewed at wileyonlinelibrary.com]

Overall, 95 787 reports mentioning at least one ICI as suspect agent were retrieved, and 650 patients (0.68%) with bone and joint injuries were found (respectively 448, 85, 39 and 78 with PD‐1, PD‐L1, CTLA‐4 inhibitors and combination therapies), accounting for 822 drug‐event pairs. Figure 3 shows the time trends of these reports; almost half of the cases (45.4%) were reported in 2019, exceeding 60% for PD‐L1 inhibitors and combination therapies (PD‐1 + CTLA‐4 inhibitors).

Disproportionality analysis was performed for 32 PTs (84 AEs were reported in less than five cases; Supplementary Table 1). Statistically significant RORs were found for eight, two and one AEs respectively with PD‐1, PD‐L1 and CTLA‐4 inhibitors, being *pathological fracture* (N = 46; ROR = 3.17; 95%CI = 2.37‐4.24), *spinal compression fracture* (42; 2.51; 1.91‐3.40) and *femoral neck fracture* (26; 2.38; 1.62‐3.50) the most common (Table 2). Mean age ranged from 54.0 ± 14.8 to 73.1 ± 9.1 years, found respectively for *fractured sacrum* and *femoral neck fracture* with PD‐1 inhibitors. All reports were serious. Latency, calculated for 44 cases without concomitant AEs, was 138 days (IQR = 48‐249.5 days).

**TABLE 2:** “Bone and joint injuries” events reported with immune checkpoint inhibitors showing statistically significant disproportionality

									Concomitant drugs				
AE	No. cases	ROR (95% CI)	Mean age	Gender	Single agent	Proportion of death	Therapeutic indications	Reporter country	Agents acting on bone resorption (proxy of osteoporosis)	Agents causing bone damage	PPIs	Endocrine IrAEs	Metastases to bone
*PD‐1 inhibitors*													
Spinal compression fracture	42	2.51 (1.91‐3.40)	70.0 ± 10.1	14 F 27 M 1 NS	PEM 18 NIV 16 IP/NIV 7 IP/PEM 1	15 (35.7%)	NSCLC 24 Melanoma 5 Thymoma 1 Other 12	AS 22 EU 9 NA 11	5 (11.9%)	9 (21.4%)	4 (9.5%)	9 (21.4%)^a^ Pancreas 5 Thyroid 4 Adrenal glands 3 Pituitary gland 2	1 (2.4%)
Pathological fracture	46	3.17 (2.37‐4.24)	64.0 ± 11.3	22 F 22 M 2 NS	NIV 30 IP/NIV 10 PEM 6	19 (41.3%)	NSCLC 14 Renal 14 NA 4 Melanoma 3 Other 11	EU 24 NA 13 SA 4 OC 2 AS 2 AF 1	1 (2.2%)	5 (10.9%)	11 (23.9%)	1 (2.2%) Thyroid 1	7 (15.2%)
Femoral neck fracture	26	2.38 (1.62‐3.50)	73.1 ± 9.1	12 F 12 M 2 NS	NIV 19 PEM 5 IP/NIV 1 PEM/NIV 1	10 (38.5%)	NSCLC 12 Melanoma 4 Other 10	AS 12 EU 10 NA 4	0 (0.0%)	2 (7.7%)	1 (3.8%)	1 (3.8%)^b^ Pituitary gland 1 Thyroid 1	0 (0.0%)
Lumbar vertebral fracture	19	2.33 (1.50‐3.62)	70.6 ± 11.6	7 F 12 M	NIV 12 PEM 5 IP/PEM 1 IP/NIV 1	5 (26.3%)	NSCLC 9 Melanoma 4 Other 6	EU 12 NA 6 AS 1	3 (15.0%)	2 (10.0%)	4 (21.1%)	3 (15.8%) Thyroid 3	0 (0.0%)
Thoracic vertebral fracture	12	2.16 (1.23‐3.81)	71.1 ± 12.3	6 F 6 M	PEM 10 NIV 2	3 (25.0%)	NSCLC 7 Melanoma 3 Other 2	EU 5 NA 3 AS 3 OC 1	0 (0.0%)	2 (16.7%)	3 (25.0%)	0 (0.0%)	0 (0.0%)
Osteoporotic fracture	11	2.86 (1.58‐5.18)	71.1 ± 7.0	4 F 7 M	NIV 5 PEM 5 IP/NIV 1	1 (9.1%)	NSCLC 4 Melanoma 4 Other 3	EU 6 NA 2 AS 2 OC 1	0 (0.0%)	1 (9.1%)	1 (9.1%)	2 (18.2%) Thyroid 1 Adrenal glands 1	0 (0.0%)
Pubis fracture	4	3.00 (1.50‐6.02)	65.3 ± 7.1	3 F 1 M	PEM 2 NIV 1 IP/NIV 1	2 (50.0%)	NSCLC 3 Melanoma 1	EU 2 AS 1 NA 1	1 (25.0%)	0 (0.0%)	1 (25.0%)	1 (25.0%) Adrenal glands 1	0 (0.0%)
Fractured sacrum	3	2.75 (1.31‐5.79)	54.0 ± 14.8	2 F 1 M	NIV 2 PEM 1	1 (33.3%)	NSCLC 2 Melanoma 1	EU 3	0 (0.0%)	1 (33.3%)	1 (33.3%)	0 (0.0%)	1 (33.3%)
*PD‐L1 inhibitors*													
Pathological fracture	8	5.48 (3.24‐9.27)	62.4 ± 16.3	1 F 7 M	ATE 7 DUR 1	2 (25.0%)	NSCLC 3 Other 5	EU 5 NA 2 SA 1	5 (62.5%)	3 (37.5%)	2 (25.0%)	0 (0.0%)	1 (12.5%)
Spinal compression fracture	9	2.77 (1.44‐5.33)	70.8 ± 9.4	5 F 4 M	ATE 7 AVE 1 DUR 1	1 (11.1%)	SCLC 2 NSCLC 2 Other 5	AS 5 NA 4	2 (22.2%)	4 (44.4%)	1 (11.1%)	3 (33.3%) Thyroid 1 Adrenal glands 1 Pituitary gland 1	1 (11.1%)
*CTLA‐4 inhibitors*													
Pathological fracture	15	3.10 (1.87‐5.15)	64.7 ± 10.3	6 F 9 M	IP/NIV 10 IP 5	5 (33.3%)	NSCLC 3 Renal 3 Melanoma 3 Other 6	NA 10 EU 3 AS 2	0 (0.0%)	3 (20.0%)	3 (20.0%)	0 (0.0%)	2 (13.3%)

Abbreviations: AE, adverse event; AF, Africa; AS, Asia; ATE, Atezolizumab; AVE, Avelumab; CI, confidence interval; DUR, Durvalumab; EU, Europe; IP, Ipilimumab; IrAEs, immune‐related adverse events; NA, North America; NIV, Nivolumab; NS, not specified; NSCLC, nonsmall cell lung cancer; OC, Oceania; PEM, Pembrolizumab; PPIs, proton pump inhibitors; ROR, reporting odds ratio; SA, South America; SCLC, small cell lung cancer.

^a^ One case with concomitant four AEs and two cases with concomitant two AEs.

^b^ Concomitant reported.

Concomitant bone metastases and endocrine irAEs were retrieved in 6.7% and 10.3% of cases, respectively. Concomitant use of drugs acting on bone resorption was reported in 8.7% of cases, being mostly found in bone fracture associated with PD‐L1 inhibitors (62.5%), while concomitant use of agents causing bone damage was retrieved in 16.4% of cases, ranging from 0.0% for pubis fracture associated with PD‐1 inhibitors to 44.4% for spinal compression fracture associated with PD‐L1 inhibitors. PPIs were concomitantly used in 16.4% of cases. Concomitant neurological AEs were retrieved in 12.8% of cases, being mostly reported in thoracic vertebral fracture associated with PD‐1 inhibitors (41.7%). *Falls* were reported in 14.4% of cases, with no significant ROR for any ICI class or combination. No case of overlapping myositis was recorded. The overall proportion of deaths was 32.8%.

## 4 DISCUSSION

To our knowledge, this is the largest comprehensive characterization of postmarketing skeletal AEs attributed to ICIs collected from a worldwide pharmacovigilance database, which allows assessment of rare events usually escaping detection/reporting in clinical trials. Only one preliminary case series has been reported so far, raising the hypothesis of skeletal events related to ICIs.^7^ Although bone‐related toxicity appears to be rare, accounting for less than 1% of overall reports, similarly to other rare irAEs (eg, hepatitis, myocarditis, endocrinopathies, severe cutaneous adverse reactions and hematological toxicities),^5^, ^6^, ^11^, ^16^, ^17^, ^18^ physicians should be aware that skeletal irAEs do occur with ICIs even in patients without other known risk factors.

Different clues emerged from our analysis: (a) bone injury can be regarded as an independent ICI‐associated irAE, considering that concomitant endocrine disorders (potentially proxy for secondary osteoporosis) were recognized only in less than 10% of cases, while myositis was never reported; (b) PD‐1 inhibitors were more frequently reported to be associated with bone toxicity, as compared to PD‐L1 and CTLA‐4 inhibitors; (c) spinal compression fractures were the most common bone irAEs reported with both anti‐PD‐1/PD‐L1 and anti CTLA‐4; (d) potential confounding clinical conditions (namely concomitant falls, bone metastases, endocrine or neurological irAEs) were reported only in a negligible proportion of cases; (e) coadministered drugs causing bone damage or acting on bone resorption were reported in limited cases; (f) skeletal lesions associated with ICIs occurred after a median of 4 to 5 months approximately, a time frame potentially consistent with skeletal remodeling and bone resorption.

Several analogies may be identified when comparing data from our pharmacovigilance analysis with the aforementioned case series,^7^ particularly age (ranging from 50 to 75 years), fracture site (vertebral column), greater involvement of PD‐1 inhibitors alone or in combination with CTLA‐4 inhibitors, types of cancer (melanoma, nonsmall cell lung cancer, renal cancer), absence of concomitant endocrine irAEs or bone metastases, and a partial overlap in time to onset of skeletal lesions (median latency of 4‐5 months in FAERS compared to 2.5‐15.5 months in our case series, and 5‐18 months in previous case series). This relatively short latency further suggests the immune‐related nature of this toxicity. Additionally, our case series highlights the occurrence of ICI‐associated bone fractures also in head and neck cancer, where neither the disease nor associated treatments are unlikely to be associated with severe osteoporosis and skeletal events.

Among coadministered drugs interfering with bone metabolism, PPIs showed the highest reporting. Given that unnecessary long‐term use of PPIs is not uncommon,^19^ deprescribing should be considered to mitigate the risk of potentially precipitating skeleton lesions in patients treated with ICIs. Additionally, a detrimental effect on ICIs efficacy due to PPIs associated gut microbiota alterations cannot be excluded.^20^

Notably, one of our patients experienced a clinical complete response after the occurrence of bone fracture. This intriguing finding should prompt further investigations to test bone toxicity as potential predictive biomarkers of a successful response to treatment with ICIs, in line with emerging evidence from other irAEs.^21^

As anticipated, biological plausibility is likely to exist, given the role of activated T cells in skeletal remodeling reported in proinflammatory states,^22^ with the production of proinflammatory cytokines and the upregulation of receptor activator of nuclear factor‐κB ligand, thus favoring osteoclasts differentiation and maturation over osteoblastogenesis.^23^ Similarly, ICI therapy activates cytokines secreting T cells, which are implicated in both tumor cell destruction and bone remodeling,^24^ thus resulting in bone loss with associated fragility and relevant risk of fractures (Figure 1).

Our analysis found an overreporting of pathological fracture with all ICIs and of osteoporotic fracture with PD‐1 inhibitors, as compared to anti‐CTLA‐4. These data could reflect the wider use of the first class of ICIs over the latter. Further investigations are needed also to assess specific risks of monotherapies vs combination regimens (including the association with other targeted therapies).

The key message of our study is that ICIs may act as precipitating factors for skeletal events. As part of dedicated close monitoring for risk stratification and early detection of skeletal lesions in patients starting treatment with ICIs, laboratory (ie, calcium/phosphorus metabolism) and imaging studies should be performed, also considering the nonnegligible impact of a fracture (ie, immobilization, high‐risk of thromboembolic events, increased operative risk) on the quality of life in advanced cancer patients. Furthermore, preexisting osteoporosis/osteopenia, genetic or environmental factors, and concomitant therapies should be carefully considered, including the assessment of body mass index (BMI), due to the potential association between sarcopenia and occurrence of irAEs.^25^ Notably, two out of four showed a BMI lower than 18.50. In this context, the implementation of dedicated guidelines for the identification, risk stratification and management of bone lesions in patients receiving ICIs should be pursued.

We acknowledge the limitations of FAERS data, in particular the inability to firmly infer a causal relationship between drug exposure and occurrence of AE.^12^ The ROR does not inform the real risk in clinical practice, mainly because of the lack of a denominator and underreporting, but only indicates an increased risk of AE reporting and not a risk of AE occurrence. Therefore, incidence rates and risk ranking cannot be derived from spontaneous reports. Furthermore, the lack of exposure data and clinical elements such as the reporting of preexisting osteopenia/osteoporosis, laboratory and radiological findings makes it difficult to fully evaluate all residual confounders involved in skeletal AEs. Notwithstanding these limitations, pharmacovigilance assessment represents an invaluable opportunity to monitor drug safety and identify novel rare signals, particularly in a setting where ethical and feasibility issues preclude actual conduction of randomized controlled trials.

## 5 CONCLUSIONS

Our large‐scale study found increased reporting of serious spinal compression fracture in patients with no apparent preexisting risk factors for skeletal injuries, thus suggesting a possible cause‐effect relationship and calling for awareness by oncologists and the implementation of dedicated guidelines. Further investigations are needed to fully characterize this novel irAE, defining patient‐ and drug‐related specific risk factors and optimal management strategies.

## CONFLICT OF INTEREST

Andrea Ardizzoni reports grants and personal fees from BMS, personal fees from MSD, Eli‐Lilly, Boehringer, and Pfzer, and grants from Celgene, outside the submitted work. Emanuel Raschi reports personal fees from Novartis, outside the submitted work. Lisa Licitra further acknowledges grant/research support from Astrazeneca, BMS, Boehringer Ingelheim, Celgene International, Debiopharm International SA, Eisai, Exelixis Inc, Hoffmann‐La Roche Ltd, IRX Therapeutics Inc, Medpace Inc, Merck‐Serono, MSD, Novartis, Pfizer, Roche, honoraria/consultation fees from Astrazeneca, Bayer, BMS, Eisai, MSD, Merck‐Serono, Boehringer Ingelheim, Novartis, Roche, Debiopharm International SA, Sobi, Ipsen, Incyte Biosciences Italy Srl, Doxa Pharma, Amgen, Nanobiotics Sa and GSK, and fees for public speaking/teaching from AccMed, Medical Science Foundation G. Lorenzini, Associazione Sinapsi, Think 2 IT, Aiom Servizi, Prime Oncology, WMA Congress Education, Fasi, DueCi promotion Srl, MI&T, Net Congress & Education, PRMA Consulting, Kura Oncology, Health & Life Srl, Immuno‐Oncology Hub. Other authors have none to declare.

## ETHICS STATEMENT

The study was approved by the Institutional Ethical Committee (INT 216/20, date of approval September 28, 2020). All patients provided written informed consent.

## Supporting information


**Supplementary Table 1**. Adverse events involving skeleton at preferred terms (PTs) level recorded with the different classes of immune checkpoint inhibitors reported in at least five cases. 95% confidence interval (CI) was calculated only for PT showing reporting odds ratio (ROR) greater than 1. Significant RORs are highlighted in bold.

## Data Availability

The data supporting the findings of this study were derived from the following resource available in the public domain: https://www.fda.gov/drugs/questions-and-answers-fdas-adverse-event-reporting-system-faers/fda-adverse-event-reporting-system-faers-public-dashboard. Further details and other data that support the findings of this study are available from the corresponding author upon request.
