# Supplementary material for: Bone fracture as a novel immune‐related adverse event with immune checkpoint inhibitors: Case series and large‐scale pharmacovigilance analysis
Source: Int J Cancer. 2021 May 4;149(3):675–83. doi: 10.1002/ijc.33592 (PMC8251715; doi:10.1002/ijc.33592)
Supplement: Supplementary file 1 — Supplementary Table 1. Adverse events involving skeleton at preferred terms (PTs) level recorded with the different classes of immune checkpoint inhibitors reported in at least five cases. 95% confidence interval (CI) was calculated only for PT showing reporting odds ratio (ROR) greater than 1. Significant RORs are highlighted in bold. [file IJC-149-675-s001.pdf]

Supplementary materials

Bone fracture as a novel immune-related adverse event with immune checkpoint inhibitors: case series and large-scale pharmacovigilance analysis

Daria Maria Filippini, Milo Gatti, Vito Di Martino, Stefano Cavalieri, Michele Fusaroli, Andrea Ardizzoni, Emanuel Raschi, Lisa Licitra

Supplementary Table 1 – Adverse events involving skeleton at preferred terms (PTs) level recorded with the different classes of immune checkpoint inhibitors reported in at least five cases. 95% confidence interval (CI) was calculated only for PT showing reporting odds ratio (ROR) greater than 1. Significant RORs are highlighted in bold.

Supplementary Table 1 – Adverse events involving skeleton at preferred terms (PTs) level recorded with the different classes of immune checkpoint inhibitors reported in at least five cases. 95% confidence interval (CI) was calculated only for PT showing reporting odds ratio (ROR) greater than 1. Significant RORs are highlighted in bold.

**Preferred terms**	**PD-1 inhibitors**		**PD-L1 inhibitors**		**CTLA-4 inhibitors**	
	**No. cases**	**ROR (95% CI)**	**No. cases**	**ROR (95% CI)**	**No. cases**	**ROR (95% CI)**
**Osteonecrosis of jaw**	36	0.60	8	0.75		
**Hip fracture**	51	0.80	7	0.63	8	0.38
**Rib fracture**	44	1.07 (0.80-1.44)	5	0.70	7	0.51
**Femur fracture**	38	0.52	8	0.62		
**Spinal fracture**	28	0.75	5	0.76	12	0.97
**Spinal compression fracture**	**47**	**2.55 (1.91-3.40)**	**9**	**2.77 (1.44-5.33)**	9	1.46 (0.76-2.81)
**Femoral neck fracture**	**26**	**2.38 (1.62-3.50)**				
**Compression fracture**	13	1.34 (0.78-2.32)				
**Lumbar vertebral fracture**	**20**	**2.33 (1.50-3.62)**				
**Thoracic vertebral fracture**	**12**	**2.16 (1.32-3.81)**				
**Cervical vertebral fracture**	7	1.06 (0.51-2.23)				
**Fractured sacrum**	**7**	**2.75 (1.31-5.79)**				
**Pelvic fracture**	7	0.45				
**Ankle fracture**	7	0.22				
**Upper limb fracture**	11	0.26			6	0.43
**Lower limb fracture**	10	0.29				
**Foot fracture**	9	0.23				
**Clavicle fracture**	12	1.57 (0.89-2.77)				
**Pubis fracture**	**8**	**3.00 (1.50-6.02)**				
**Joint injury**	9	0.24				
**Osteonecrosis**	21	0.33	5	0.45	6	0.29
**Joint dislocation**	9	0.43				
**Osteoporotic fracture**	**11**	**2.86 (1.58-5.18)**				
**Pathological fracture**	**46**	**3.17 (2.37-4.24)**	**14**	**5.48 (3.24-9.27)**	**15**	**3.10 (1.87-5.15)**
**Pelvic fracture**	7	0.45				
**Facial bones fracture**	8	0.87				
**Bursitis**	19	0.87			5	0.69
**Rotator cuff syndrome**	9	0.32				
**Humerus fracture**	16	1.54 (0.94-2.51)				
